# Independent infections of porcine deltacoronavirus among Haitian children

**DOI:** 10.1038/s41586-021-04111-z

**Published:** 2021-11-17

**Authors:** John A. Lednicky, Massimiliano S. Tagliamonte, Sarah K. White, Maha A. Elbadry, Md. Mahbubul Alam, Caroline J. Stephenson, Tania S. Bonny, Julia C. Loeb, Taina Telisma, Sonese Chavannes, David A. Ostrov, Carla Mavian, Valery Madsen Beau De Rochars, Marco Salemi, J. Glenn Morris

**Affiliations:** 1 Emerging Pathogens Institute, University of Florida, Gainesville, FL USA; 2 Department of Environmental and Global Health, College of Public Health and Health Professions, University of Florida, Gainesville, FL USA; 3 Department of Pathology, Immunology and Laboratory Medicine, College of Medicine, University of Florida, Gainesville, FL USA; 4 Christianville Foundation, Gressier, Haiti; 5 Department of Health Services Research, Management and Policy, College of Public Health and Health Professions, University of Florida, Gainesville, FL USA; 6 Department of Medicine, College of Medicine, University of Florida, Gainesville, FL USA

**Keywords:** Computational biology and bioinformatics, SARS-CoV-2

## Abstract

Coronaviruses have caused three major epidemics since 2003, including the ongoing SARS-CoV-2 pandemic. In each case, the emergence of coronavirus in our species has been associated with zoonotic transmissions from animal reservoirs^1,2^, underscoring how prone such pathogens are to spill over and adapt to new species. Among the four recognized genera of the family *Coronaviridae*, human infections reported so far have been limited to alphacoronaviruses and betacoronaviruses^3–5^. Here we identify porcine deltacoronavirus strains in plasma samples of three Haitian children with acute undifferentiated febrile illness. Genomic and evolutionary analyses reveal that human infections were the result of at least two independent zoonoses of distinct viral lineages that acquired the same mutational signature in the genes encoding Nsp15 and the spike glycoprotein. In particular, structural analysis predicts that one of the changes in the spike S1 subunit, which contains the receptor-binding domain, may affect the flexibility of the protein and its binding to the host cell receptor. Our findings highlight the potential for evolutionary change and adaptation leading to human infections by coronaviruses outside of the previously recognized human-associated coronavirus groups, particularly in settings where there may be close human–animal contact.

## Abstract (web summary)

The presence of porcine deltacoronavirus has been detected in three children from Haiti that could have originated from zoonotic spillover.

## Main

Coronaviruses are enveloped, positive-sense single-stranded RNA viruses that belong to the family *Coronaviridae*. Porcine deltacoronavirus (PDCoV) is a member of the genus *Deltacoronavirus*, and was reported for the first time in Hong Kong, China, in 2012 (refs. ^6,7^). PDCoV causes gastrointestinal symptoms in piglets, with dehydration and possibly death^8^. The jejunum and ileum are the primary sites of infection^8–11^. There are reports of symptomatic infection, in experimental settings, in chickens, turkeys and calves^12,13^. Human cells have also been reported to be permissive to PDCoV infection^14^. Binding of the PDCoV spike glycoprotein to an interspecies conserved site, the host aminopeptidase N^14–17^, may facilitate direct transmission to non-reservoir species, possibly including humans.

Haiti is part of the island of Hispaniola, and one of the poorest countries in the world^18^. The local pig population was wiped out in the 1980s to eliminate African swine fever from the area^19^, followed by subsequent and ongoing repopulation from North America, Europe and China^19,20^. Pig farming in the country is at a subsistence level, and, to our knowledge, no cases of PDCoV infection have been reported to date in pigs. Between 2012 and 2020, our group monitored the occurrence of illness among children seen at a free school clinic operated by the Christianville Foundation school system in the Gressier region of Haiti^21–24^. Respiratory and diarrhoeal illnesses were most common among children presenting to the clinic for care, followed by acute undifferentiated febrile illnesses (that is, fever with no clear localizing symptoms), which accounted for approximately 16% of clinic cases^21^.

## Detection of Hu-PDCoV

As part of ongoing studies at the Christianville school clinic, we collected plasma samples from 369 children with acute undifferentiated febrile illness seen at the clinic between May 2014 and December 2015. As previously reported^25^, all children from whom samples were collected were screened for common pathogens associated with fever, including malaria and viral pathogens such as dengue, Zika and Chikungunya viruses. Plasma samples that were negative in these assays were then cultured in Vero E6 cells, as a ‘non-biased’ means of identifying potential new or emerging viruses. Cultures of three samples (0.8% of the 369 samples collected) were positive for coronavirus strains, which clustered with PDCoV. Information on the children from whom these three samples were obtained is provided in Table 1.

**Table 1:** Sample collection date and epidemiology of children with PDCoV infection

Child	Plasma no.	Collection date	Age, gender	School	PDCoV strain	GenBank no.	GenBank no. of closest PDCoV strain by BLAST analysis
1	0081-4	December 2014	7 years, female	A	Haiti/human/0081-4/2014	MW685622	KY065120 (pig/China/2016)
2	0256-1	March 2015	7 years, female	B	Haiti/human/0256-1/2015	MW685623	KR150443 (pig/USA/2015)
3	0329-4	April 2015	6 years, male	A	Haiti/human/0329-4/2015	MW685624	KY065120 (pig/China/2016)

Cases 1 and 3 (samples 0081-4 and 0329-4, respectively) were in-patients from the main campus of the school (school A) that is attended by students from semi-urban areas. This school provides classes for grades K–13 (ages 5–20); the socioeconomic status of families of students ranges widely, with a subset of families coming from low-income families and receiving tuition support. Case 2 (sample 0256-1) was from a different campus (school B), which is an elementary school located in the mountains approximately 1-h drive from school A; the school is in a rural area, with students from very low socioeconomic backgrounds. All three children presented with a history of fever but recovered uneventfully: child 2 was febrile (40 °C) when seen in the clinic; child 1 and child 3 reported cough and abdominal pain. Although they reported a fever, child 3 did not have acute symptoms when seen in the clinic.

**Fig. 1:**
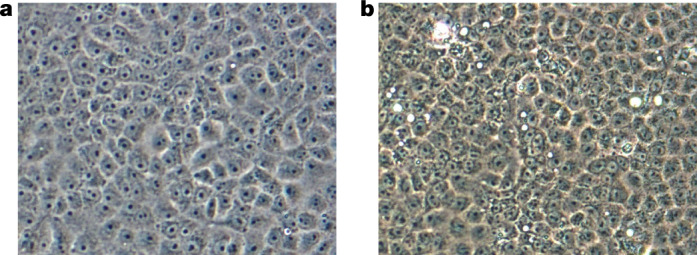
Non-specific CPEs formed by plasma from patient 0081-4 in Vero E6 cells. **a**, Mock-infected Vero E6 cells, 11 days post-inoculation with phosphate-buffered saline. **b**, Vero E6 cells 11 days post-inoculation with plasma from patient 0081-4. Original magnification, ×200.

Nucleic acids purified as previously described^26,27^ from the plasma samples of the three children tested negative for alphavirus and flavivirus RNAs^26,27^ by PCR with reverse transcription (RT–PCR). Virus isolation was also attempted after inoculation of aliquots of the plasma onto Vero E6 cells^26,27^. Nucleic acids, purified from the cell culture medium 7, 14, 21 and 30 days post-inoculation of the cells, again tested negative for alphavirus and flavivirus RNAs. Moreover, they tested negative for the DNA and RNA of common human respiratory viruses using a GenMark Respiratory Panel^28^. However, subtle cytopathic effects (CPEs) were observed in Vero E6 cell monolayers starting at about 11 days post-inoculation, suggesting that a virus had been isolated. The non-specific CPEs included granulation of the cells (Fig. 1).

**Extended Data Fig. 1:**
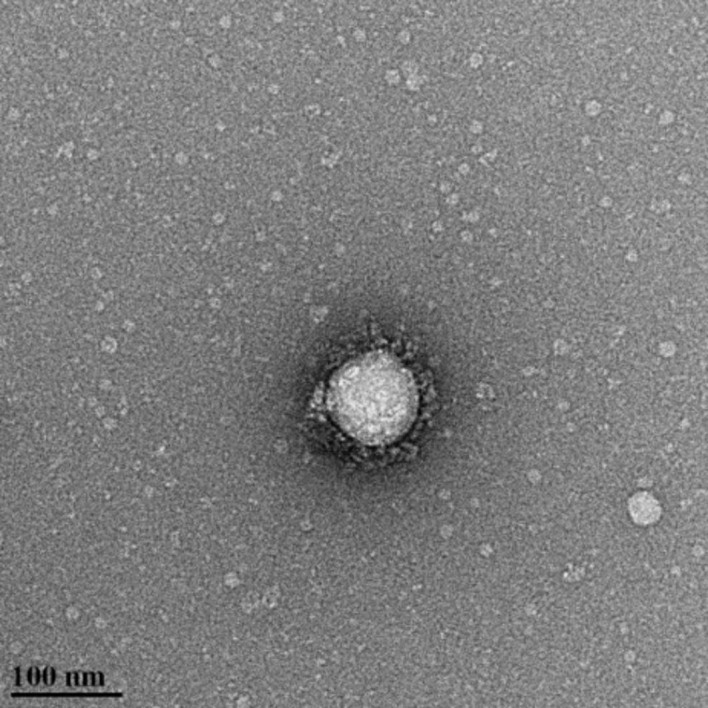
Negative stained coronavirus particle viewed by transmission electron microscopy. Typical coronavirus spikes rim the spherical virus particle.

Because none of the tests produced evidence that could be used for a preliminary identification of a viral agent, an unbiased amplification and sequencing approach^29^ was attempted for cells inoculated with plasma from sample 0081-4, which displayed more CPEs than cells inoculated with the other two plasma samples. PCR amplification yielded seven amplicons. Sequence analyses indicated that six amplicons were African green monkey sequences from the Vero E6 cells, whereas one 401-bp amplicon had 100% identity with the genome sequence of various PDCoV strains. Therefore, RNA purified from Vero E6 culture samples was retested using a pan-coronavirus RT–PCR test that amplifies a conserved 668-bp region within the RNA-dependent RNA polymerase gene of alphacoronaviruses, betacoronaviruses gammacoronaviruses and deltacoronaviruses^30^, generating positive results. A 3-ml sample of cell culture medium collected from Vero E6 cells that displayed CPEs 11 days post-inoculation was subsequently screened by transmission electron microscopy. Rare spherical and pleomorphic coronavirus particles that ranged from 90 to 120 nm in diameter were visualized with negative staining. A representative image of a 110-nm spherical coronavirus particle is shown in Extended Data Fig. 1.

A follow-up test using RNA directly purified from plasma generated the same 668-bp amplicons, providing further indications that PDCoV was present in the plasma and cell culture samples. Mock-inoculated Vero E6 cultures and 20 additional randomly selected plasma samples from the study tested negative for PDCoV RNAs. At the time, we had no PDCoV strains in our laboratory. Following this preliminary identification, whole-genome sequences for the three isolates were obtained by Sanger sequencing. The GenBank accession numbers corresponding to the sequenced genomes are provided in Table 1.

## Genomic analyses of Hu-PDCoV strains

**Extended Data Fig. 2:**
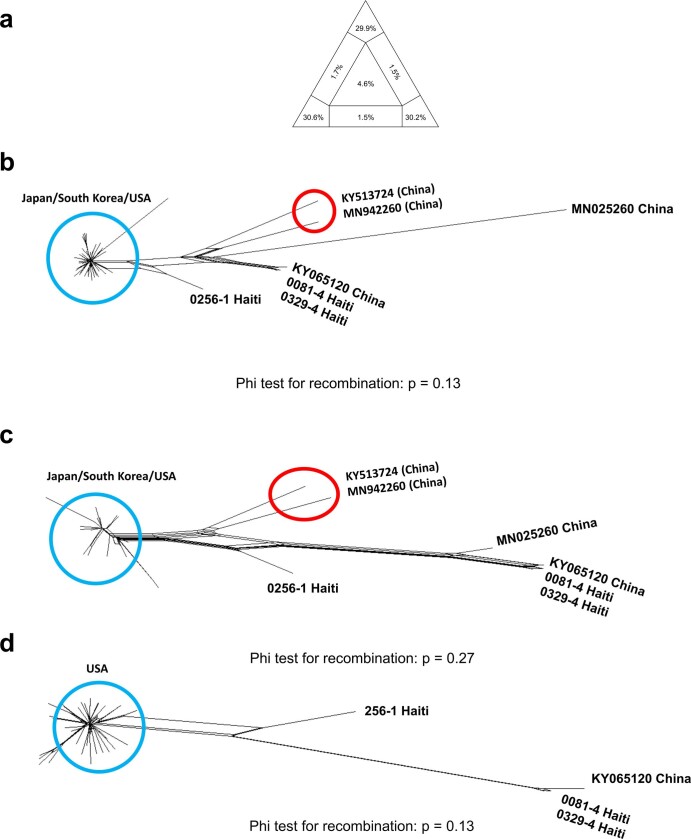
Assessment of phylogenetic signal and Neighbor net plot of PDCoV fragments. **a**, Likelihood mapping of the final 109 sequences alignment (see Methods) showing extremely low phylogenetic noise (4.6% in the center of the triangle), as required for reliable phylogeny inference. **b**, Neighbor net inferred from pair-wise p-distances of 47 genomes for the major genome fragment identified by RDP4 (see Methods). Recombination signal was assessed by the PHI test. No significant evidence of recombination was found in either fragment (PHI test p > 0.05). **c**, Neighbor net and PHI test for the minor genome fragment identified by RDP4 (see Methods). The only change in topology, in the split decomposition networks shown in panels **a** and **b**, concerned two Chinese sequences (highlighted by the red circle) displaying mixed ancestry possibly due to homoplasy. These sequences were also removed before performing any further phylogenetic analyses. **d**, Neighbor net and PHI test for the full genome alignment only including Haitian Hu-PDCoV strains and closest Chinese and American strains (after removal recombinant strains detected by RDP4).

In agreement with previous reports^31^, assessment of potential recombinants in the multiple sequence alignment including all currently available full-genome PDCoV sequences detected a signal for recombination. Recombination events involved 60 strains from pigs in China belonging to sequence clusters unrelated to the new human isolates. After all recombinant strains were removed, the pairwise homoplasy index (PHI)^47^ test for recombination using the alignment of the remaining full genomes, as well as the recombinant fragments identified in the sequences from China, did not show any recombination signal (*P* > 0.05). Moreover, NeighborNets inferred from full genomes, as well as recombinant fragments, showed the human sequences from Haitian individuals consistently clustering with non-recombinant porcine strains of Chinese (child 1 and child 3) or US (child 2) origin (Extended Data Fig. 2). The human PDCoV (Hu-PDCoV) strains 0081-4 and 0329-4, identified 4 months apart in child 1 and child 3, respectively, while attending school A (Table 1), were highly similar (99.97%), and closely related (99.8%) to a pig strain detected 1 year later in Tianjin, China. Child 2, who attended school B, was infected with a variant, 0256-1, closely related to a pig strain detected in Arkansas, USA, in 2015.

**Extended Data Fig. 3:**
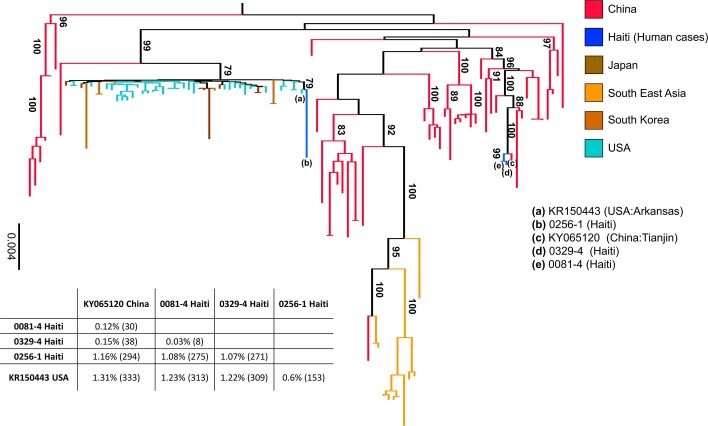
Maximum likelihood tree of PDCoV strains. The tree was inferred from 109 PDCoV full genome sequences, including four sparrow CoV genomes for outgroup rooting (accession numbers MG812375, MG812376, MG812377, and MG812378, from Chen et al.^44^) using the best fitting nucleotide substitution model (see Methods). For display purposes, outgroup sequences were removed from the figure. Vertical branches are scaled in number of nucleotide substitutions per site according to the bar on the left of the tree. Bootstrap values > 75% (1000 replicates) are indicated along supported branches. The table at the bottom of the tree shows nucleotide % dissimilarity (and total number of nucleotide differences in parenthesis) between Haitian strains and their closest non-Haitian relatives, which are labelled in tree by progressive letters according to the legend on the right.

Since recombination did not affect the new Hu-PDCoV strains nor their closest evolutionary relatives, we inferred a maximum likelihood tree from all full-genome PDCoV sequences available to investigate in more detail the origin of the human isolates. The tree clearly shows that strains 0081-4 and 0329-4, and strain 0256-1 belong, respectively, to two distinct and well-supported monophyletic clades: the first clade clustering strains from pigs in China and the second clade clustering strains from pigs in the USA (Extended Data Fig. 3). It is important to emphasize that while phylogenetic relationships in the deep branches of the maximum likelihood tree cannot be considered an accurate depiction of the evolutionary relationships among major PDCoV clades because of recombination, clustering within the two clades including the strains detected in Haiti is not affected by recombinant events (Extended Data Fig. 2). The three PDCoV sequences identified in the Haitian children were the result of at least two separate zoonotic transmissions from related non-recombinant porcine strains that probably occurred within a similar time frame. There are two possible scenarios that could explain why two Haitian children were infected with genetically similar PDCoV strains. There could have been two independent zoonoses from animals infected with highly genetically similar viruses. Alternatively, there could have been one initial zoonosis followed by human-to-human transmission. Since samples from pigs in the areas surrounding the two schools were not available, it is impossible at this time to discern which scenario is the most likely. Regardless, the phylogeny demonstrates the occurrence of two distinct PDCoV lineages in school A and school B, highlighting the ability of deltacoronaviruses to spill over successfully in the human population.

**Fig. 2:**
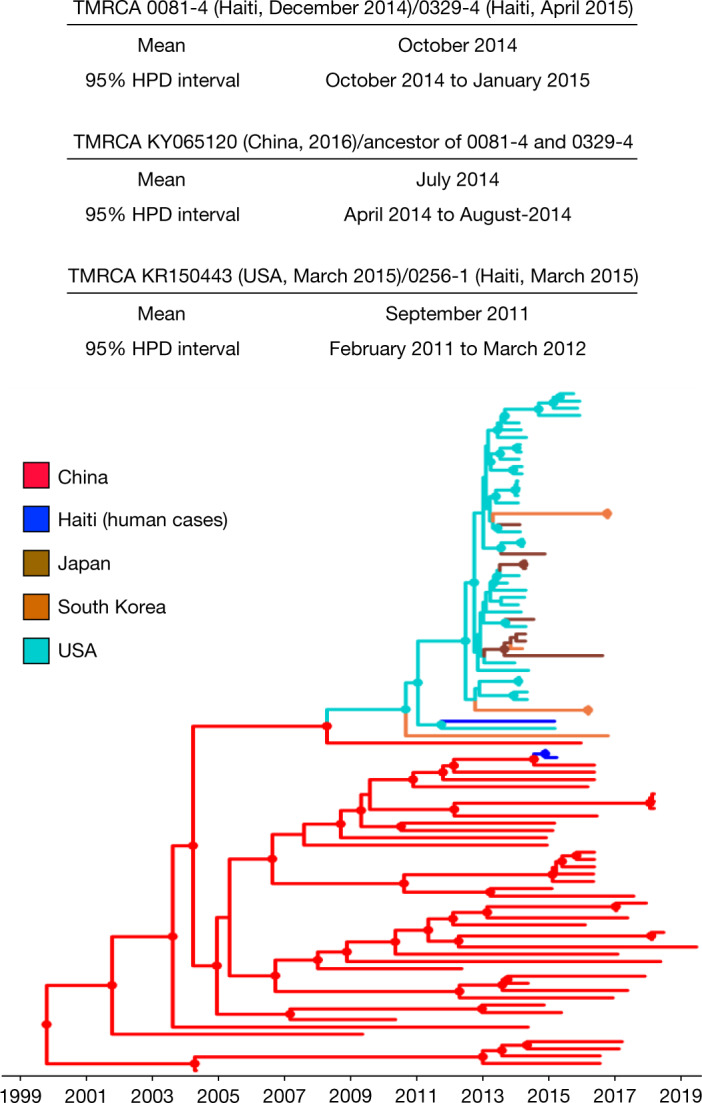
Bayesian maximum clade credibility tree of PDCoV strains. The circles at the internal nodes indicate high posterior probability support of more than 0.9. The branch lengths were scaled in time by using a strict molecular clock. The information above the tree shows the inferred TMRCA between strains discovered in Haiti and their closest phylogenetic relative, with 95% HPD intervals.

**Extended Data Fig. 4:**
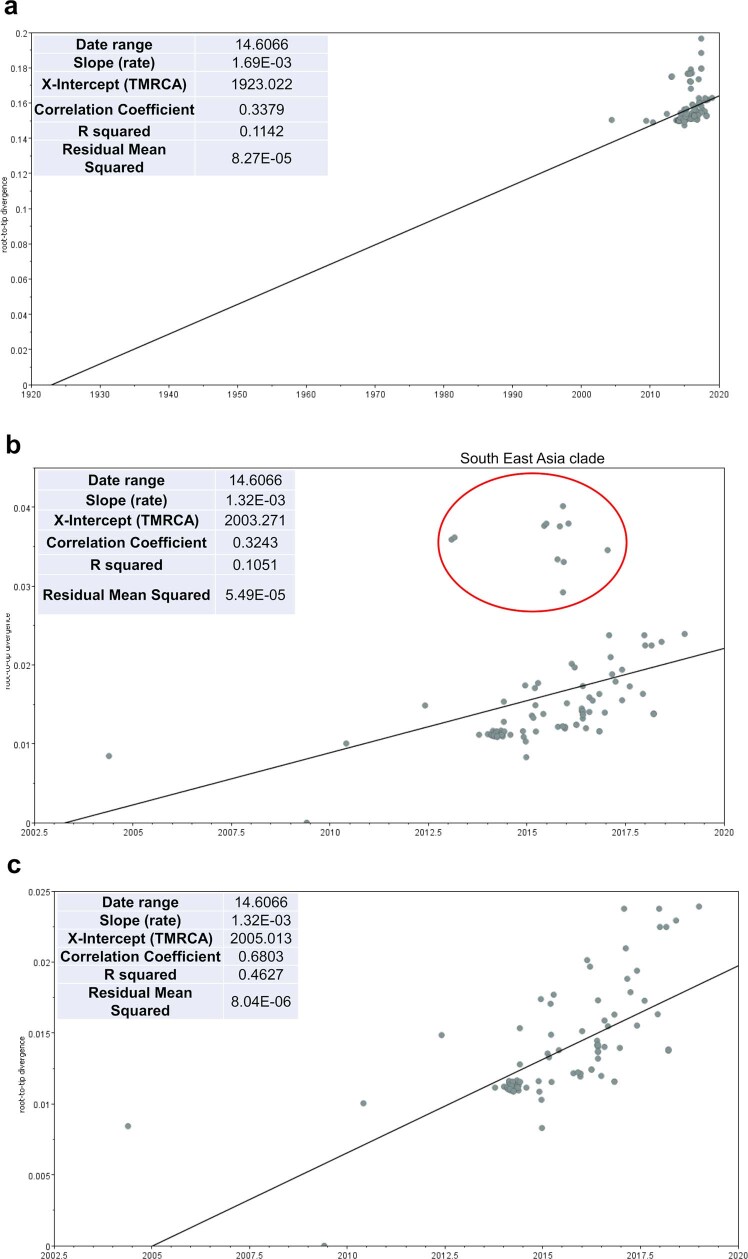
Analysis of the temporal signal with TempEst. **a**, **b**, Root-to-tip distance (y-axis) *vs*. sampling time linear regression in the ML likelihood phylogeny inferred from: all PDCoV sequences in the final data set (n = 109 sequences) including sparrow outgroup sequences (**a**); PDCoV sequences without outgroups (n = 105 sequences), with sequences in the red circle belonging to the South East Asia clade (see Fig. 1) showing a clear departure from the strict molecular clock model (**b**); and PDCoV sequences after removal of the South East Asia clade (n = 94 sequences), showing greatly improved clock signal (R^2^ = 0.68) (**c**).

**Extended Data Fig. 5:**
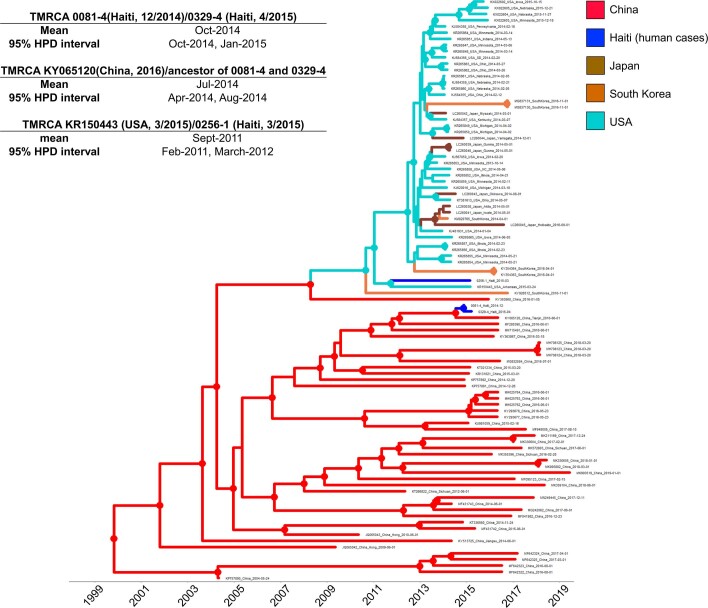
Bayesian maximum clade credibility (MCC) tree of PDCoV strains. The MCC tree was evolutionary rate. Circles at internal nodes indicate high posterior probability (PP) support >0.9. The table on inferred from a subset of 94 full genome strains that displayed sufficient temporal signal for molecular clock calibration. Branch lengths were scaled in time, according to the bar at the bottom, by using a strict molecular clock and sampling dates to estimate PDCoV the left shows the inferred time of the most recent common ancestor (TMRCA) between Haitian strains and their closest phylogenetic relative, with 95% high posterior density intervals (95%HPD).

Our next step was the calibration of a molecular clock to infer the time of the most recent ancestor (TMRCA) of Hu-PDCoV and their most closely related porcine strains. We tested for the presence of a temporal signal in the sequence dataset by calculating the linear regression between root-to-tip distances and sampling time in the maximum likelihood tree. After removal of sparrow outgroup sequences and the southeast Asian clade outliers, the tree inferred from the remaining (*n* = 94) sequences showed sufficient signal to calibrate a molecular clock (Extended Data Fig. 4). The topology of the Bayesian maximum clade credibility tree obtained using a strict molecular clock confirmed the findings of the maximum likelihood phylogeny (Fig. 2, Extended Data Fig. 5). Identical results were obtained with the relaxed clock model. The mean evolutionary rate estimated with the strict clock resulted in 7.3 × 10^−4^ nucleotide substitutions per site per year, with a 95% high posterior density (HPD) interval of 5–9 × 10^−4^, which is slightly higher than previous estimates but with overlapping confidence intervals^32^. According to the clock calibration, 0081-4 and 0329-4 TMRCA dates to October 2014, with 95% HPD intervals essentially overlapping (October 2014 to January 2015) with the sampling dates (see Table 1) of the strains themselves. In turn, the strains detected in Haiti diverged from their MRCA with the pig strain detected in China in July 2014 (95% HPD: April to August 2014). Conversely, the isolate 0256-1 TMRCA discovered in Haiti and the isolate KR150443 detected in the USA date to 2011 (95% HPD: February 2011 to March 2012). It is possible that PDCoV strains had been circulating in pigs in Haiti for a few years, as also suggested by its relatively long terminal branch in the maximum likelihood tree (Extended Data Fig. 3), before infecting the human patient and that we are missing several intermediate links along the 0256-1 branch, either from pig or other human strains. Unfortunately, given the paucity of detailed information of recent livestock importations into Haiti as well as the lack of a surveillance system to monitor viral infections in farm animals, a detailed reconstruction of the events surrounding introduction of PDCoV into Haiti and its subsequent introduction into humans is currently not possible.

**Fig. 3:**
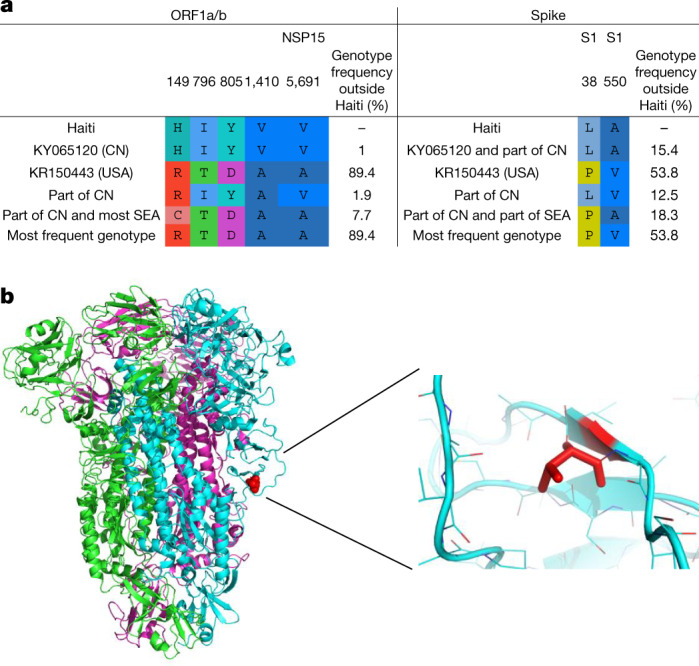
Analysis of conserved amino acids in human PDCoV strains. Residues are numbered based on the ORF1a/b absolute amino acid position in the reference sequence JQ065043 (pig/China). **a**, Amino acid signature pattern analysis on ORF1a/b. CN, China; SEA, southeast Asia. **b**, The trimer structure of the spike glycoprotein. Different colours are assigned to each monomer: red indicates residue 550, where Haitian sequences have a valine to alanine mutation.

Although the Hu-PDCoV strains belong to independent evolutionary lineages, introduced in humans through what would appear to be at least two separate zoonotic transmissions, a more in-depth analysis of the genomic changes shows that the three strains detected in Haiti share a signature of five conserved amino acid residues in the ORF1a/b polyprotein and two in the spike glycoprotein, unique among other currently known PDCoV sequences from pigs (Fig. 3a). The sole exception is the strain KY065120 discovered in China, which displays the same amino acid signature and is the one most closely related to the strains discovered in Haiti from school A (Table 1, Extended Data Fig. 3) and may represent a porcine strain pre-adapted for effective transmission to humans. Indeed, the convergent evolution of identical amino acid changes along distinct phylogenetic lineages is highly suggestive of an adaptive response. Mutations in the first five ORF1a/b amino acids that are part of the Hu-PDCoV-specific signature (Fig. 3a) are located at sites that do not correspond to solved crystal structures. The other ORF1a/b mutation maps in non-structural protein 15 (Nsp15): A30V (the amino acid position is numbered according to the reference sequence JQ065043). The carboxy-terminal domain of the Nsp15 protein possesses endoribonuclease with uridylate-specific activity^33^. Although the protein is not necessary for RNA synthesis, it is necessary in coronaviruses to escape recognition of double-stranded RNA intermediates by the host^34^. PDCoV NS15 inhibits the induction of interferon-β, the main intestinal antiviral cytokine, by preventing nuclear translocation of the interferon regulatory factor IRF1 (ref. ^35^). The last two mutations in the Hu-PDCoV-specific signature map in the spike glycoprotein. The first mutation, P8A, in the amino-terminal domain of the glycoprotein, is not resolved in the known crystallographic structure, possibly because the segment is too flexible to be seen by cryo-electron microscopy. The second mutation, V550A, is located in the S1 subunit (between the receptor-binding domain and the cleavage site between S1 and S2) on a short β-sheet forming intramolecular contact with a neighbouring loop (Fig. 3b). The V550A change observed in Hu-PDCoV (removal of two methyl groups) is present at relatively low frequency in other PDCoVs discovered in Asia, neither of which displays the additional amino acid changes observed in the strains detected in Haiti. This change, albeit minor, eliminates specific Van der Waals contact (with proline at position 535 and the backbone carbonyl at position 532), potentially enhancing protein flexibility and dynamic movement of S1. Since mutations that prevent intermolecular spike protein interactions between S1 and S2 of the SARS-CoV-2 variant B.1.1.7 have been observed (A570D, D614G and S982A)^36,37^, V550A may represent a common mechanism that enhances dynamic movements, accelerating membrane fusion events and transmission of the virus.

## Conclusions

To our knowledge, this is the first report of PDCoV infection in humans, consistent with viraemia and systemic dissemination. The recent divergence of human strains detected in Haiti from their closest pig strains detected in China and the USA in the phylogeny highlights how little we know about the spreading of PDCoV and its introduction in Haiti. Recent data regarding the movements of live pigs and meat into the country are lacking^38^, and movements of pigs and their pathogens across the globe can be unexpectedly complicated and difficult to track^39^, stressing the need for further studies. Our findings, however, are consistent with a virus maintained in the swine population and is capable of successful spillover in humans. Children infected with PDCoV only had mild illness, with less than 1% acute undifferentiated febrile illness during the time period studied, suggesting that the strains identified do not represent a major human health threat. We would emphasize, however, that this study only identified symptomatic children who were acutely viraemic with PDCoV. Further serological studies will be needed to identify the frequency with which such infections occur in the general Haitian population, with the caution that serological studies may be difficult because of possible cross-reactivity with human endemic coronaviruses. Nonetheless, our data highlight the potential for PDCoV zoonoses into human populations, especially in rural or less-developed regions where contact with domestic animals is common.

## Methods

### Clinical sample collection

From 2012 to 2020, our research group monitored a cohort of approximately 1,250 school children attending one of four schools in the Christianville Foundation school system in the Gressier region of Haiti^21^. Children attending schools in this school system had free access to medical care through a school-based clinic. The study was approved by the Institutional Review Board (IRB) at the University of Florida and the Haitian National IRB; written informed consent for sample collection was obtained from parents of participants, with assent from participants. For this study, children presenting to the school clinic between May 2014 and December 2015 with an acute undifferentiated febrile illness, defined as a history of fever and/or a measured temperature over 37.5 °C in the clinic with no localizing symptoms or signs (that is, no respiratory, skin, or urinary symptoms or signs) were invited to enrol^25^. After enrolment, clinic healthcare providers recorded clinical data in a study questionnaire and a sample of venous blood (1–3 ml) was collected in an acid citrate dextrose blood collection tube. The blood samples were subsequently centrifuged to pellet the platelets, red blood cells and white blood cells, and the resulting plasma was aseptically transferred to cryovials and stored at −80 °C for subsequent analysis. Appropriate medical care on the basis of clinical presentation and laboratory studies was provided to study participants by clinic healthcare providers. Data on the identification of arboviruses and other virus species among children participating in the study have been previously reported^22,23,25–27,40,41^. As the study was done in young children, the amount of plasma collected was limited, and samples have, in most instances, been exhausted, owing to the range of studies initially conducted on the samples while screening for other pathogens. IRB restrictions limit our ability to share samples outside of our institution.

### Cell culture lines

The African green monkey kidney cell line Vero E6 (Vero C1008) was obtained from the American Type Culture Collection (ATCC; catalogue no. CRL-1586), which authenticates the cells they sell. Upon culture, the cells displayed epithelial morphology (as expected). Before the preparation of seed stocks, the cells were treated for 3 weeks with plasmocin, then verified free of mycoplasma DNA by PCR using a Takara Bio USA mycoplasma detection kit.

### Virus identification and sequencing

Attempts at next-generation sequencing using an Illumina MiSeq platform generated minimal coverage, so we sorted to Sanger sequencing using the primer system outlined by Liang et al.^42^, with one addition: to obtain the 5′ ends of the viral genomes, a rapid amplification of cDNA ends (RACE) kit was used per the manufacturer’s protocols (Life Technologies), and the resulting amplicons were TA-cloned into plasmids and sequenced. PCR amplicons for Sanger sequencing were amplified using AccuScript High-Fidelity reverse transcriptase (Agilent Technologies) in the presence of SUPERase-In RNase inhibitor (Ambion), followed by PCR with Q5 DNA polymerase (New England Biolabs). They were next purified using a QIAquick PCR purification kit (Qiagen) before TA cloning. The inserts in the plasmids were subsequently sequenced bidirectionally using a gene-walking approach, on the basis of obtaining at least 800 bp or non-ambiguous sequence. Briefly, pairs of non-overlapping primers and Q5 polymerase were used to produce 42 separate amplicons corresponding to the PDCoV genome, and each amplicon was Sanger sequenced bidirectionally.

### Transmission electron microscopy

For visualization of negative-stained PDCoV by transmission electron microscopy (TEM), 3 ml of cell culture medium that had been harvested from Vero E6 cells that displayed subtle CPE 11 days post-inoculation with plasma was concentrated to approximately 200 µl since it was anticipated that the virus yield would be low and electron microscopy evaluation would be time-consuming. The cell medium was concentrated using an Amicon Ultra-15 centrifugal filter unit that has an Ultracel-100 membrane with a molecular mass cut-off of 100 kDa (Millipore). This was accomplished through centrifugation at 4,000*g* for 10 min at room temperature until the retentate had a volume of around 200 μl, after which it was recovered and transferred to a sterile cryovial. Thereafter, 100 μl of the retentate was mixed with an equal volume of freshly prepared 2% paraformaldehyde in 0.1 M PBS (pH 7.20) in preparation for TEM. Thereafter, PCOV was visualized by TEM after an aliquot of the fixed sample was negatively stained at the UF Interdisciplinary Center for Biotechnology Research (ICBR) Electron Microscopy Laboratory (RRID: SCR_019146). For negative staining, a glow-discharged 400-mesh carbon-coated Formvar copper grid was floated on a 5 μl aliquot of virus suspension for 5 min, then washed twice with water. Excess solution was drawn off with filter paper, and the grid floated on 1% (w/v) aqueous uranyl acetate for 30 s. Excess stain was removed with filter paper, the grid was air dried and then examined using a FEI Tecnai G2 F20-TWIN transmission electron microscope (FEI Corporation) that was operated at 200 kV, with digital images acquired using a 4k × 4k CCD camera and Digital Micrograph software (Gatan). Other grids prepared in the same manner were also examined with a FEI Tecnai G2 Spirit Twin transmission electron microscope and digital images were acquired with a Gatan UltraScan 2k × 2k camera and Digital Micrograph software.

### Sequence data assembly

The identity of the whole-genome sequences was confirmed via BLAST^43^ of the nr/nt NCBI database. Following positive identification, available PDCoV sequences from pigs were downloaded from the NCBI (www.ncbi.nlm.nih.gov), together with closely related sparrow deltacoronavirus sequences^44^ (Supplementary Table 1) to be used as outgroups in the phylogenetic analysis (see below).

The final full-genome dataset assembled included 104 PDCoV genomes from pigs, 4 from sparrows (Supplementary Table 1) and 3 newly sequenced Hu-PDCoV strains, which were aligned with MAFFT v.7.407 (ref. ^45^). A potential recombinant origin of Haitian sequences was assessed using the NeighborNet algorithm^46^, an algorithm based on the PHI test, as implemented in Splitstree^47,48^ v.4.14.8, and with the RDP4 (ref. ^49^) software package v.4.97.

### Phylogenetic and amino acid signature analysis

A phylogenetic signal was verified using likelihood mapping^50^ (Extended Data Fig. 2), as implemented in IQTREE v.2.0.6 (ref. ^51^). A maximum likelihood tree was calculated using the same version of IQTREE, with the best-fitting nucleotide substitution model according to the Bayesian information criterion and 1,000 bootstrap replicates. The correlation between root-to-tip genetic divergence and sampling dates to assess the clock signal of the alignment was performed with TempEst^52^ before Bayesian phylodynamic analyses. The time-scaled tree was calculated using the Bayesian phylodynamic inference framework in BEAST v1.10.4 (ref. ^53^). Markov chain Monte Carlo samplers were run for 200 million generations, with sampling every 20,000 generations, to ensure proper mixing, which was assessed by calculating the effective sampling size of each parameter estimate. The HKY nucleotide substitution model was used with empirical base frequencies and gamma distributions of site-specific rate heterogeneity^54^. The molecular clock was calibrated with a strict clock choosing either a constant size or a Bayesian Skyline Plot demographic prior^55^. A maximum clade credibility tree was inferred from the posterior distribution of trees using TreeAnnotator, specifying a burn-in of 104 million and median node heights. The maximum clade credibility tree was edited graphically using ggtree^56–58^. Markov chain Monte Carlo runs with different demographic priors gave the same result. An analysis with the relaxed molecular clock resulted in a nearly identical mean rate estimate and did not show a rate significantly different from the mean along any of the branches of the tree (coefficient of variation 95% HPD including zero), including the branches leading to the Hu-PDCoV isolates.

Signature pattern analysis of the strains discovered in Haiti compared with the rest of the downloaded sequences was performed with the online version of VESPA^59^; the PDCoV reference sequence JQ065043.2 was used as a guide for the codon coordinates. The spike glycoprotein three-dimensional structure (Protein Data Bank ID: 6B7N)^60^ was used as a base, and figures were generated using PyMol^61^.

### Reporting summary

Further information on research design is available in the Nature Research Reporting Summary linked to this paper.

## Online content

Any methods, additional references, Nature Research reporting summaries, source data, extended data, supplementary information, acknowledgements, peer review information; details of author contributions and competing interests; and statements of data and code availability are available at 10.1038/s41586-021-04111-z.

## Supplementary information

Supplementary Table 1 Accession numbers of deltacoronaviruses accessed from NCBI.

Reporting Summary

## Data Availability

The GenBank accession numbers for sequence data are included in Table 1. Supplementary Table 1 includes a list of the accession numbers of deltacoronaviruses accessed from NCBI for the phylogenetic studies.
