# Supplementary material for: Independent infections of porcine deltacoronavirus among Haitian children
Source: Nature. 2021 Nov 17;600(7887):133–7. doi: 10.1038/s41586-021-04111-z (PMC8636265; doi:10.1038/s41586-021-04111-z)
Supplement: Supplementary file 1 — Accession numbers of deltacoronaviruses accessed from NCBI. [file 41586_2021_4111_MOESM1_ESM.pdf]

nature portfolio

https://doi.org/10.1038/s41586-021-04111-z

Supplementary information

Independent infections of porcine deltacoronavirus among Haitian children

In the format provided by the authors and unedited

NCBI Accession Number	Country of Origin
JQ065042	China
JQ065043	China
KJ481931	USA
KJ567050	USA
KJ584355	USA
KJ584356	USA
KJ584357	USA
KJ584358	USA
KJ584359	USA
KJ620016	USA
KM820765	SouthKorea
KP757890	China
KP757891	China
KP757892	China
KR131621	China
KR150443	USA
KR265847	USA
KR265848	USA
KR265849	USA
KR265850	USA
KR265851	USA
KR265852	USA
KR265853	USA
KR265854	USA
KR265855	USA
KR265856	USA
KR265857	USA
KR265858	USA
KR265859	USA
KR265860	USA
KR265861	USA
KR265862	USA
KR265863	USA
KR265864	USA
KR265865	USA
KT021234	China
KT266822	China
KT336560	China
KT381613	USA
KU051641	Thailand
KU051649	Thailand
KU981059	China
KU984334	Thailand
KX022602	USA

KX022603	USA
KX022604	USA
KX022605	USA
KX118627	Laos
KX361343	Thailand
KX361344	Thailand
KX361345	Thailand
KX834351	Vietnam
KX834352	Vietnam
KX998969	Vietnam
KY065120	China
KY293677	China
KY293678	China
KY354363	SouthKorea
KY354364	SouthKorea
KY363867	China
KY363868	China
KY513724	China
KY513725	China
KY926512	SouthKorea
LC260038	Japan
LC260039	Japan
LC260040	Japan
LC260041	Japan
LC260042	Japan
LC260043	Japan
LC260044	Japan
LC260045	Japan
MF041982	China
MF095123	China
MF280390	China
MF431742	China
MF431743	China
MF642322	China
MF642323	China
MF642324	China
MF642325	China
MF948005	China
MG242062	China
MG812375	USA
MG812376	USA
MG812377	USA
MG812378	USA
MG832584	China
MG837130	SouthKorea

MG837131	SouthKorea
MH025762	China
MH025763	China
MH025764	China
MH708123	China
MH708124	China
MH708125	China
MH715491	China
MK005882	China
MK211169	China
MK330604	China
MK330605	China
MK355396	China
MK359104	China
MK572803	China
MK993519	China
MN025260	China
MN249445	China
MN942260	China
