# Supplementary material for: Independent infections of porcine deltacoronavirus among Haitian children
Source: Nature. 2021 Nov 17;600(7887):133–7. doi: 10.1038/s41586-021-04111-z (PMC8636265; doi:10.1038/s41586-021-04111-z)
Supplement: Supplementary file 2 — Reporting Summary [file 41586_2021_4111_MOESM2_ESM.pdf]

nature research

Corresponding author(s): *Double-blind peer review submissions: write DBPR and your manuscript number here instead of author names.*

Last updated by author(s): *YYYY-MM-DD*

Reporting Summary

Nature Research wishes to improve the reproducibility of the work that we publish. This form provides structure for consistency and transparency in reporting. For further information on Nature Research policies, see our Editorial Policies and the Editorial Policy Checklist.

Statistics

For all statistical analyses, confirm that the following items are present in the figure legend, table legend, main text, or Methods section.

n/a	Confirmed	
☐	☒	The exact sample size (*n*) for each experimental group/condition, given as a discrete number and unit of measurement
☐	☒	A statement on whether measurements were taken from distinct samples or whether the same sample was measured repeatedly
☐	☒	The statistical test(s) used AND whether they are one- or two-sided<br>*Only common tests should be described solely by name; describe more complex techniques in the Methods section.*
☒	☐	A description of all covariates tested
☒	☐	A description of any assumptions or corrections, such as tests of normality and adjustment for multiple comparisons
☒	☐	A full description of the statistical parameters including central tendency (e.g. means) or other basic estimates (e.g. regression coefficient) AND variation (e.g. standard deviation) or associated estimates of uncertainty (e.g. confidence intervals)
☒	☐	For null hypothesis testing, the test statistic (e.g. *F*, *t*, *r*) with confidence intervals, effect sizes, degrees of freedom and *P* value noted<br>*Give P values as exact values whenever suitable.*
☐	☒	For Bayesian analysis, information on the choice of priors and Markov chain Monte Carlo settings
☐	☒	For hierarchical and complex designs, identification of the appropriate level for tests and full reporting of outcomes
☒	☐	Estimates of effect sizes (e.g. Cohen's *d*, Pearson's *r*), indicating how they were calculated

Our web collection on statistics for biologists contains articles on many of the points above.

Software and code

Policy information about availability of computer code

Data collection	All software was commercially obtained; details and sources are included in the manuscript
Data analysis	Specified in manuscript

For manuscripts utilizing custom algorithms or software that are central to the research but not yet described in published literature, software must be made available to editors and reviewers. We strongly encourage code deposition in a community repository (e.g. GitHub). See the Nature Research guidelines for submitting code & software for further information.

Data

Policy information about availability of data

All manuscripts must include a data availability statement. This statement should provide the following information, where applicable:

- Accession codes, unique identifiers, or web links for publicly available datasets
- A list of figures that have associated raw data
- A description of any restrictions on data availability

All data are included in the manuscript. Personal identifiers are protected by IRB and HIPAA regulations.

Field-specific reporting

Please select the one below that is the best fit for your research. If you are not sure, read the appropriate sections before making your selection.

☒ Life sciences ☐ Behavioural & social sciences ☐ Ecological, evolutionary & environmental sciences

For a reference copy of the document with all sections, see nature.com/documents/nr-reporting-summary-flat.pdf

Life sciences study design

All studies must disclose on these points even when the disclosure is negative.

Sample size	Observational study; all enrolled participants included in the described studies
Data exclusions	None
Replication	Original plasma samples were collected from children, and of necessity were small volume; samples available for these studies were extremely limited, as initial aliquots of each sample were used for screening for other pathogens. We were unable to perform repeat measures due to the lack of available sample material. An attempt to obtain the genetic sequences of the viruses using NGS on an Illumina MiSeq was unsuccessful, presumably because the amount of PDCoV vRNA was very low; these runs provided minimal coverage and only short segments of the viral genomes were recovered. The MiSeq platform requires Cq values of ≤ 20 for adequate coverage for sequencing. As described in the manuscript, whole genome sequences for the three isolates were obtained by Sanger sequencing.
Randomization	Observational study
Blinding	Observational study

Reporting for specific materials, systems and methods

We require information from authors about some types of materials, experimental systems and methods used in many studies. Here, indicate whether each material, system or method listed is relevant to your study. If you are not sure if a list item applies to your research, read the appropriate section before selecting a response.

Materials & experimental systems

n/a	Involved in the study	
☒	☐	Antibodies
☐	☒	Eukaryotic cell lines
☒	☐	Palaeontology and archaeology
☒	☐	Animals and other organisms
☐	☒	Human research participants
☐	☒	Clinical data
☒	☐	Dual use research of concern

Methods

n/a	Involved in the study	
☒	☐	ChIP-seq
☒	☐	Flow cytometry
☒	☐	MRI-based neuroimaging

Eukaryotic cell lines

Policy information about cell lines

Cell line source(s)	The African green monkey kidney cell line Vero E6 (Vero C1008) was obtained from the American Type Culture Collection (ATCC, Manassas, VA, USA, catalog. no. CRL-1586), which authenticates the cells they sell. Upon culture, the cells displayed epithelial morphology (as expected).
Authentication	see above
Mycoplasma contamination	Prior to the preparation of seed stocks, the cells were treated for 3 weeks with plasmocin, then verified free of mycoplasma DNA by PCR using a Takara Bio USA mycoplasma detection kit
Commonly misidentified lines (See ICLAC register)	*Name any commonly misidentified cell lines used in the study and provide a rationale for their use.*

Human research participants

Policy information about studies involving human research participants

Population characteristics	Study participants were in grades Pre-K to 13, of both genders, and had presented for care to the school clinic associated

Population characteristics	with the Christianville school system in Gressier, Haiti. Details on the specific study participants included in this manuscript are in Table 1.
Recruitment	All students presenting to the Christianville school clinic with complaints of undifferentiated febrile illness were invited to enroll in the study.
Ethics oversight	The study was approved by the University of Florida IRB and the Haitian National IRB. Ethics training and oversight was provided by Dr. Madsen Beau de Rochars, a University of Florida faculty member and Haitian national and a co-author on the study.

Note that full information on the approval of the study protocol must also be provided in the manuscript.

Clinical data

Policy information about clinical studies

All manuscripts should comply with the ICMJE guidelines for publication of clinical research and a completed CONSORT checklist must be included with all submissions.

Clinical trial registration	Observational study; not a clinical trial
Study protocol	*Note where the full trial protocol can be accessed OR if not available, explain why.*
Data collection	*Describe the settings and locales of data collection, noting the time periods of recruitment and data collection.*
Outcomes	*Describe how you pre-defined primary and secondary outcome measures and how you assessed these measures.*
